# Genomic Insights into the Fungal Pathogens of the Genus *Pneumocystis*: Obligate Biotrophs of Humans and Other Mammals

**DOI:** 10.1371/journal.ppat.1004425

**Published:** 2014-11-06

**Authors:** Philippe M. Hauser

**Affiliations:** Institute of Microbiology, Centre Hospitalier Universitaire Vaudois and University of Lausanne, Lausanne, Switzerland; Duke University Medical Center, United States of America

*Pneumocystis* organisms were first believed to be a single protozoan species able to colonize the lungs of all mammals. Subsequently, genetic analyses revealed their affiliation to the fungal Taphrinomycotina subphylum of the Ascomycota, a clade which encompasses plant pathogens and free-living yeasts. It also appeared that, despite their similar morphological appearance, these fungi constitute a family of relatively divergent species, each with a strict specificity for a unique mammalian species [1]. The species colonizing human lungs, *Pneumocystis jirovecii*, can turn into an opportunistic pathogen that causes *Pneumocystis* pneumonia (PCP) in immunocompromised individuals, a disease which may be fatal. Although the incidence of PCP diminished in the 1990s thanks to prophylaxis and antiretroviral therapy, PCP is nowadays the second-most-frequent, life-threatening, invasive fungal infection worldwide, with an estimated number of cases per year above 400,000 [2]. Despite this clinical importance, studies of *P. jirovecii* progressed slowly, at least in part because of the lack of a continuous culture system. Nevertheless, recent genomic findings provided insights into the lifestyle of these fungi.

## Genome Sequences

Despite the absence of culture, the nuclear genome sequences of three *Pneumocystis* spp. have been recently released to the public. That of *P. carinii*, the species infecting rats, was obtained from resected lungs of an infected rat and purification of *P. carinii* cells (pgp.cchmc.org). This provided sufficient amounts of relatively pure DNA for conventional cloning and sequencing of chromosomes separated on gel, as well as for high throughput DNA sequencings (HTS). The nuclear genome sequence of *P. murina*, the species infecting mice, was obtained from resected lungs followed by HTS (*Pneumocystis murina* Sequencing Project, Broad Institute of Harvard and the Massachusetts Institute of Technology, http://www.broadinstitute.org/). Thanks to cell immunoprecipitation and whole genome amplification, the *P. jirovecii* nuclear genome sequence was obtained using HTS from a single bronchoalveolar lavage fluid sample of a single patient with PCP [3]. Because of the low DNA purity, the assembly necessitated an innovative approach using iterative identification of the fungal homologies among the reads from the host and lung microbiota. The genome sequences of the mitochondria were also obtained from the same sequencings, as well as by PCR [4], [5].

## Genomic Assemblies

The nuclear genome assembly of *P. carinii* is still highly fragmented, whereas that of *P. murina* is made of 17 contigs likely to correspond to the 17 chromosomes composing this genome (Table 1). The *P. jirovecii* nuclear assembly presents an intermediary fragmentation, which results from the difficulty of assembling it out of a mixture of reads. Because of their repetitive nature, telomeres were not assembled. The nuclear genomes of the three *Pneumocystis* spp. are approximately 8 Mb.

**Table 1:** Statistics of *Pneumocystis* spp. nuclear and mitochondrial genomic assemblies^a^.

	*P. jirovecii*	*P. murina*	*P. carinii*
**Nuclear**			
Assembly size [Mb]^b^	8.1	7.45	6.3
Contigs	358	17	4'278
N50 [kb]	41.6	491	2.2
Mean GC content [%]	28.4	26.9	32.5
Protein coding genes^c^	3'898	3'557	4'591
Coding regions [%]	68.9	66.3	50.0
rRNA operon	1	1	1
tRNA genes	77	47	36^d^
**Mitochondrial**			
Assembly size [kb]	35.1	24.6	26
Structure	circular	linear	linear
Contigs	1	1	1
GC content [%]	25.7	29.8	29.8
Protein coding genes	14	14	14
Orfs (unknown function)	1	2	4
rRNA genes	2	2	2
tRNA genes	25	28	25

a The data of the nuclear genomes are from Cissé et al. [3], except those of *P. murina* which are from broadinstitute.org. The data of the mitochondrial genomes are from Sesterhenn et al. [4] and Ma et al. [5].

b The nuclear assemblies of the three species are considered to be incomplete and do not include repetitive elements.

c The numbers for *P. jirovecii* and *P. carinii* overestimate proteome size because some peptides are from incomplete coding DNA sequences at ends of contigs.

d Only complete copies in *P. carinii* assembly were considered.

The mitochondrial genomes of *P. murina* and *P. carinii* are approximately 25 kb, whereas that of *P. jirovecii* is around 35 kb (Table 1). This size difference is due to a supplementary region that is non-coding and highly variable in size and sequence among isolates [5]. The circular structure of the *P. jirovecii* mitochondrial genome differs from the linear one present in the two other *Pneumocystis* spp. The biological significance of this difference remains unknown.

## Genome Content

Using gene models specifically developed, the three *Pneumocystis* spp. nuclear genomes were predicted to encode approximately 3,600 protein coding genes (Table 1). Mapping of the genes onto the chromosomes is achieved for *P. murina* because contigs correspond to chromosomes, as well as for *P. carinii* because isolated chromosomes were sequenced, but not for *P. jirovecii*. Functional annotation was optimized by the use of transcription data as well as of carefully chosen fungal proteomes as intermediary data for mapping onto the Kyoto Encyclopedia of Genes and Genomes (KEGG) atlas of biochemical pathways [3], [6]. About 30%–40% of the genes were reported to encode hypothetical proteins without significant homology with the databases, but this proportion is decreasing as new fungal genome sequences are released. A maximum likelihood phylogeny from the alignment of 458 concatenated orthologs revealed *Taphrina deformans* and the members of the *Schizosaccharomyces* genus as the closest relatives of *Pneumocystis* spp. [3], [7]. The genome content of the three *Pneumocystis* spp. covered most of the biochemical pathways corresponding to the basal metabolism and standard cellular processes [6], [8]. Specific features included the presence of a single operon encoding the ribosomal RNA (Table 1), such as *T. deformans* [7], which contrasts with the tens or hundreds in other fungi, and the lack of common fungal virulence factors such as the glyoxylate cycle and polyketide synthase clusters [3], [6], [8]. The mitochondrial genomes of the three *Pneumocystis* spp. encode 15 to 17 proteins (Table 1).

## Genomic Insights by Comparative Genomics

Relevant features of the *P. carinii* genome were compared to those of the free-living yeast *S. cerevisiae* and of the extreme fungal obligate parasite *Encephalitozoon cuniculi* [9]. Intermediate values of gene number, genome size, and mean intergenic space suggested that *P. carinii* might be in the process of becoming dependent on its host. The use of *Schizosaccharomyces pombe* genome sequence as a control for genomic annotation revealed the absence of most of the enzymes specifically dedicated to the synthesis of amino acids in *Pneumocystis* spp. [3], [6]. Whole genome comparison to other representatives of the Taphrinomycotina subphylum revealed several supplementary gene losses in *P. carinii* and *P. jirovecii* (*P. murina* could not be analyzed because its genome sequence was released prior to publication under specific terms). The hypothetical gene repertoires of ancestors were reconstructed using maximum parsimony and the irreversible Dollo model of evolution [10]. The approach identified approximately 2,000 genes presumably lost by the common ancestor of *Pneumocystis* and *Taphrina* genera during its evolution towards the *Pneumocystis* genus. Analysis of these genes revealed losses of genes that impair the biosynthesis of thiamine, the assimilation of inorganic nitrogen and sulfur, and the catabolism of purines. In addition, lytic proteases, which are believed to be crucial to fungal virulence, were underrepresented. The absence of the genes was ascertained by extensive gene searches in partially overlapping data sets expected to cover 100% of the genomes, and by the fact that it was observed in both *P. carinii* and *P. jirovecii*. Nevertheless, it is important to keep in mind that we cannot firmly exclude the presence of genes of a previously unknown origin that have not been observed in other organisms so far, and thus would be undetectable because they are absent from the databases. These gene losses constitute an important signature of the lifestyle of *Pneumocystis* spp.

## Obligate Parasitism

The loss of biosynthetic pathways of essential molecules is a hallmark of obligate parasitism in both eukaryotic and prokaryotic organisms [11], [12]. These losses are believed to be allowed by the availability of the end products of the pathways within the host environment. Similarly, the absence of substrate may explain the loss of assimilation pathways. The loss of amino acids and thiamine biosyntheses in *Pneumocystis* spp. strongly suggests that they are obligate parasites. Thus, their entire cycle probably takes place within the host lungs, and no free-living form of these fungi would exist. In addition to amino acids and thiamine, *Pneumocystis* spp. are believed to scavenge cholesterol from their host to build their own membranes [13]. In bacteria, gene losses associated with obligate parasitism imply a reduction of the genome size linked to a reduction of the guanine-cytosine (GC) content [14]. Likewise, *Pneumocystis* spp. genomes have smaller genome size and GC content than their free-living and facultative parasite relatives, respectively *Schizosaccharomyces* spp. and *T. deformans* (Table 2).

**Table 2:** Some features of selected microorganisms with various lifestyles.

Kingdom	Lifestyle	Organism	Genome size (Mb)	% GC	Minimum missing pathways	Reference
Fungi	Obligate animal biotroph	*Pneumocystis* spp.	8	30	amino acids synthesis	3, 10
					thiamine synthesis	
					nitrogen+sulfur assimilation	
					purine degradation	
	Obligate animal parasite	*Encephalitozoon cuniculi*	3	47	amino acids synthesis	6
					vitamins synthesis	
					nucleotides synthesis	
					lipids synthesis	
	Free-living	*Saccharomyces cerevisiae*	13	38	-	6
		*Schizosaccharomyces* spp.	14	36	nitrogen+sulfur assimilation	10
	Facultative plant biotrophs	*Taphrina deformans*	13	50	-	7
		*Ustilago maydis*	20.5	54	-	16
	Obligate plant biotrophs	*Blumeria graminis*	120	44	thiamine synthesis	16
					nitrogen+sulfur assimilation	
		*Golovinomyces orontii*	160	50	thiamine synthesis	16
					nitrogen+sulfur assimilation	
		*Puccinia graminis*	89	46	thiamine synthesis	17
					nitrogen+sulfur assimilation	
Protista	Obligate animal parasite	*Plasmodium falciparum*	23	21	amino acids synthesis	18
		*Leishmania major*	33	55	amino acids synthesis	18
		*Cryptosporidium hominis*	10	32	amino acids synthesis	18

## Biotrophy

Two categories of parasites are recognized: biotrophs, which secrete low amounts of lytic enzymes and obtain food from living host cells, and necrotrophs, which secrete many degrading enzymes and toxins and obtain food from dead host cells [15]. The missing pathways of *Pneumocystis* spp. are shown in Table 2 together with those of selected microorganisms with various lifestyles. The requirement in thiamine and the absence of inorganic nitrogen and sulfur assimilation are hallmarks of obligate plant biotrophs [16]. Several other biological characteristics of *Pneumocystis* spp. are also hallmarks of obligate biotrophs: (i) the absence of destruction of host cells during colonization as well as during pathogenic infection, (ii) the lack of known virulence factors, (iii) a sex life cycle occurring within the host, and (iv) the difficulty to be cultured in vitro so far [8], [16], [17]. On the other hand, the loss of the catabolism of purines and of the amino acids syntheses revealed in *Pneumocystis* spp. has not been observed in fungal biotrophs so far. The first loss might be specific to *Pneumocystis* spp., but the second is a hallmark of organisms feeding on other animals, such as obligate parasites of humans [18] (Table 2). This latter loss might be related to the adaptation to animal hosts and suggests that there are more proteins available in animal than plant hosts. Thus, the genomic and biological characteristics of *Pneumocystis* spp. suggest the working hypothesis that they are obligate biotrophic parasites of mammals. This hypothesis has been already proposed previously on the basis of the analysis of *P. carinii* transcriptome [8], as well as of the biological characteristics of *Pneumocystis* spp. [1]. Experiments are required in order to test this hypothesis. In particular, the computational predictions supporting obligate biotrophy need to be verified. *Pneumocystis* spp. would be the first fungal biotrophs of animals recognized so far. Of note, the lifestyle of *P. jirovecii* differs from the other fungal obligate parasites of humans, *Malassezia* and *Candida* spp., which are obligate commensals and opportunistic necrotrophs.

The reduction of genome size in *Pneumocystis* spp. contrasts with the increase of this parameter in some fungal obligate plant biotrophs (Table 2). This latter increase corresponds to a proliferation of retrotransposons, which may create genetic variability and diversity including panels of effectors involved in virulence [16]. As in *Ustilago maydis* (Table 2), the genome size reduction in *Pneumocystis* spp. might be compensated by the genetic diversity generated by sexuality, suggesting that they might be obligate sexual organisms. This hypothesis would fit the fact that the asci issued from the sexual cycle might be the unique airborne particles responsible for the transmission of the fungus [19].

## Epidemiological Consequence

Obligate parasitism of *P. jirovecii* has important implications in the management of immunocompromised patients susceptible to PCP. Indeed, the fungus is most probably restricted to the lungs of humans so that the only sources of the infection are patients with PCP and colonized humans, such as infants experiencing primo-infection and transitory carriers, as well as, possibly, pregnant women and elderly people [20].
